# The relationship between serum vitamin D level and wheezing and allergy

**DOI:** 10.1186/1939-4551-8-S1-A66

**Published:** 2015-04-08

**Authors:** Derya Ufuk AltSntaş, Umut Mansuroğlu, Gülbin Bingöl Karakoç, Dilek Karagöz, Mustafa Yimaz

**Affiliations:** 1 Çukurova University ,Department of Pediatrics, Division of Allergy and Immunology, 01140, Adana, Turkey

## Background

In this study, the factors that affect the vitamin D level on healthy children and the relationship between Vitamin D and wheezing, allergy, infection has been researched.

## Methods

This study has been planned in parallel with a birth cohort study which was done to 1377 children who were born in Adana between February 2010- 2011. The babies’ vitamin D level was examined from their blood serums and atopic status at the beginning and the end of the study. Half of the babies have been recommended to continue to take 400 units vitamin D daily .

## Results

The age average of the babies who were studied was 20,3± 1,5 months. 46,9 % of the babies were girls, 53,1 % were boys. At the beginning of the study 12,5 % of the babies’ serum 25(OH)D_3_ levels were deficient, 39,1 % of the babies’ were insufficient, 48,4 % of the babies’ were sufficient. The average of the babies’ vitamin D level was 29,8 ±10,8 ng/ml. At the end of study, 29,7 % of the babies’ serum 25(OH)D_3_ levels were sufficient, 45,3% of the babies’ were insufficient, 25% of the babies’ were deficient. At the beginning of the study 55% of the babies’ who had at least one wheezing, serum _25(OH)D3_levels were insufficient or deficient. Compared to the babies who did not have wheezing, the difference was statically meaningful (p:0,04). The 71,4 % of the babies’ who had allergy, serum 25(OH)D_3_ levels were insufficient or deficient but a statically meaningful relationship was not detected. The end of the study 81,1% of the babies’ who had at least one wheezing, serum 25(OH)D_3_ levels were insufficient or deficient . That was statically meaningful (p:0,02). The 81,2% of the babies’ who had allergy, serum 25(OH)D_3_ levels were insufficient or deficient. While a meaningful relationship between vitamin D intake and wheezing and infection was not found, at the end of the study slightly meaningful relationship between vitamin D intake and allergy was detected.

At the end of study, 29,7 % of the babies’ serum 25(OH)D_3_ levels were sufficient, 45,3% of the babies’ were insufficient, 25 % of the babies’ were deficient.

## Conclusions

In this study, meaningful relationship between vitamin D and wheezing, and slightly meaningful relationship with allergic diseases was detected. Still, to be able to find out vitamin D’s effect on allergy and wheezing, advanced studies which are supported by genetic examinations and more patients are needed.

